# Facilitators of professional socialisation of learners in the clinical learning areas: A qualitative systematic review

**DOI:** 10.4102/curationis.v45i1.2172

**Published:** 2022-02-17

**Authors:** Julia L. Mafumo, Azwidihwi R. Tshililo, Takalani R. Luhalima

**Affiliations:** 1 Department of Advanced Nursing Science, Faculty of Health Sciences, University of Venda, Thohoyandou, South Africa

**Keywords:** professional socialisation, learner nurses, professional nurses, clinical learning environment, role model

## Abstract

**Background:**

Professional socialisation is significant in nursing as it involves immersing learners in the profession so that they adopt the ethical values and conduct of the profession. It is in the clinical learning areas where learners observe and practise those values. The objective of the review was to explore the factors that promote professional socialisation of learners in the clinical learning areas. The problem is the inadequate support for learner nurses in the clinical learning areas. The South African community has lost trust in nurses and that was even acknowledged in the national nursing summit in 2011.

**Objectives:**

To present a review of the factors that facilitate professional socialisation among undergraduate nursing learners.

**Method:**

A systematic review was conducted on literature from 2008 to 2018. The literature search focused on factors that facilitate professional socialisation of learner nurses. A search of databases was conducted in CINAHL, MEDLINE, Google Scholar and Science Direct. The search focused on literature on professional socialisation of learner nurses published from 2008 to 2018. The search resulted in 3035 articles which were further reduced to 13 after further synthesis. Critical appraisal skills programme was used to assess the quality of the studies.

**Results:**

Three main themes emerged. Learner factors, factors in the clinical learning areas and interpersonal factors were identified as the factors that facilitate professional socialisation of learners.

**Conclusion:**

Learners should have self-motivation and be supported and assisted to develop a positive professional identity. The clinical learning environment should have effective communication that fosters learning. Professional nurses should act as exemplary role models so that learners can emulate the conduct and practice. The review brought to light that the professional socialisation of learners is affected by the learner factors, clinical learning areas and personal factors.

## Introduction

Professional socialisation is the process whereby a person who is new in the profession adopts the norms and values of the profession to make them his/hers and act as such. Dinmohammadi, Peyrovi and Mehrdad (2017) defined professional socialisation as a process whereby the student develops and internalises a professional identity through the acquisition of knowledge, skills, attitudes, beliefs, values, norms and ethical standards in order to fulfil a professional role. Professional socialisation in nursing is significant in ensuring that learners are supported in adopting the values of the profession (De Swardt, Van Rensburg & Oosthuizen 2017). These values are highly recognised in the profession as they guide and motivate practitioners in their interactions with patients, colleagues and other professionals (Bimray, Jooste & Julie 2019).

The journey to professional socialisation is not a smooth process as one must suddenly adopt and adapt to a certain way of behaviour and conduct. Professional socialisation happens through professional identity which is complex and can only be met through socialisation and conformity to the expectations of the organisation (Cruess, Cruess & Steinert 2019). In nursing, the development of professional identity is difficult because of its relationship with the personal identity, gender, past experiences, professional knowledge, social networks and students’ relationships and interactions with the academic institutions (Ewertsson et al. 2017; Neishabouri, Ahmadi & Kazemnejad 2017). Hence, the support of the clinical staff is important to assist learners in professional socialisation.

### Problem statement

In the past few years unethical and inappropriate conduct by the nurses has been reported in the media where nurses are portrayed as uncaring and incompetent (Kagan et al. 2015). In those reports, nurses were labelled as lacking basic principles of caring including compassion and empathy, patients often being ignored and their needs not attended to in time (Johannesen, Hovland & Steen 2014; McAllister et al. 2014). Unethical and unacceptable conduct by trained staff negatively impacts on the professional socialisation of learners.

The professional nurses’ misconduct and unethical behaviour is a problem rampant worldwide. A study in Canada found that from 2012 to 2014, the cases of professional nurses’ misconduct have been increasing (Tomaszewski et al. 2016). Professional nurses were found guilty of misconduct ranging from theft and fraud to failure in providing adequate care (Tomaszewski et al. 2016). The South African Nursing Council’s statistical report on misconduct cases for nurses for the period of March 2018 to October 2018 indicates that seven nurses in two provinces were charged for misconducts including poor nursing care, assault on patients, maternity-related and medication-related frauds (SANC 2018). These misconducts could be attributed to the poor professional socialisation during training and pracrtice as professional norms and values are learnt and internalised during professional socialisation. If these values are not reinforced during professional socialisation, it is less likely that the person will have them after completion. Hence, the researcher needed to identify the factors that facilitate professional socialisation of learners in the clinical learning areas. Leaner nurses are socialised to behave in a professionally accepted manner through interaction with clinical staff in the clinical areas. During the interaction, professional conduct and attributes are consciously and unconsciously transferred to learner nurses. These professional attributes are expected to be acquired before the completion of training, and continue throughout their practice. If during professional socialisation, learners are exposed to unethical conduct and behaviours from the professional nurses, they are more likely to be involved in professional misconduct as indicated in Bandura’s Social Learning Theory (David 2015).

### Literature review

Professional socialisation of learner nurses involves teaching of nursing ethics and values to learners. This takes place in both the theoretical and clinical areas of their training (De Swardt et al. 2017). It is in the clinical learning areas where learners come into the real world when they integrate what they have learnt in theory into practice (Lee & Yang 2019). This is where professional socialisation takes place through interaction with patients and hospital staff. Learners would be properly socialised in a relaxed environment as learning is effective when one is in a good space. At times learning in the clinical area could be challenging, constantly changing, unpredictable and stressful (Bimray et al. 2019). For effective professional socialisation, the clinical learning environment should have good communication and interaction in order to promote safe patient care wherein learners would feel free to learn (Flott & Linden 2016).

The people who are entrusted with the responsibility to promote interaction and communication are the professional nurses as they are the ones who are in charge of the learning of students in the clinical area. In nursing education standards, the *Nursing Act (33, 2005)* indicated that professional nurses are responsible for the clinical supervision of learners. Mutual respect between the clinical staff and learners is the key ingredient in professional socialisation as it allows the learners to explore the impact of clinical learning and they can learn in a relaxed environment. This was confirmed by a study in Malaysia which indicated that there was a positive association between providing support and encouragement to learners, and the learners’ performance in the clinical learning areas (Ludin & Fathulla 2016). In another study in Malawi, Bvumbwe, Malema and Chipeta (2015) found that the acquisition of competency in learners is influenced by the quality of and interactions between the clinical staff and learners, thereby indicating that where there is a constructive clinical environment with adequate opportunities, the development of competency is higher.

De Swardt et al. (2017) in their study in Gauteng province indicated that a positive clinical environment where there is proper supervision, leadership and good interpersonal relationships could lead to learners becoming competent nurse practitioners who would be able to provide quality nursing care. The learners must also play an integral part in professional socialisation where they have an intrinsic motivation that drives them to want to achieve and excel in their learning. A positive attitude of the learner is also imperative as this will assist in developing a positive professional identity which is the first step to professional socialisation (Neishabouri et al. 2017). The nursing education institutions must play an essential role in the clinical learning areas by providing support and communication regarding factors that promote learning and professional socialisation (Onuoha, Prescott & Daniel 2016).

### Aim

The objective of the study was to present a review of the literature on factors that facilitate professional socialisation of undergraduate nursing learners in the clinical learning environment. The review attempts to answer the following questions: (1) What are the factors in the clinical learning areas that facilitate professional socialisation for undergraduate nursing learners? (2) What role do professional nurses play in the professional socialisation of learners? The results were used to identify factors in the clinical learning areas that impact professional socialisation among undergraduate nursing learners in an attempt to effectively socialise these learners in the nursing profession.

### Research method and design

A systematic review was used to obtain data from the reviewed literature. Systematic review is research synthesis conducted to identify and retrieve international evidence relevant to a question/s and to appraise and synthesise the results (Munn et al. 2018). Systematic literature review is also viewed as a means of summarising and presenting overviews of findings which could be current and historically derived from primary sources including published literature, and papers cited in peer review process (Aromataris & Pearson 2014). The review dwells on factors that facilitate the professional socialisation of learners in the clinical learning environment.

## Methods

### Search strategy

Literature was extensively searched along a wide range of primary research to come out with comprehensive information (Aromataris & Pearson 2014). An extensive systematic search of databases for this review was conducted in Ebcohost, CINAHL, MEDLINE, Google Scholar and Science Direct. The search focused on factors that promote professional socialisation of learner nurses in the clinical learning areas. The following keywords were used to retrieve relevant literature for this review: ‘professional socialisation’ or ‘professional development in the clinical learning areas’, ‘student nurse’ OR ‘learner nurse and professional socialisation in nursing practice’, ‘professional nurse’ OR ‘registered nurse role in professional socialisation of learners in the clinical areas’.

### The inclusion and exclusion criteria

The inclusion criteria identified for this review were qualitative full-text articles on professional socialisation published from 2008 to 2018, research undertaken with undergraduate nursing students on professional socialisation of learners and written in English. The researcher used the indicated 10-year period, which was viewed as adequate time to get enough data. The population in the reviewed literature was undergraduate nursing students and professional nurses who were responsible for the professional socialisation of learners.

The search was also limited to journal articles excluding books, newspaper articles and conference papers. The other exclusion criteria for the review were quantitative articles, mixed methods research work, research undertaken with allied health sciences students and those that were not written in English. The quantitative and mixed-method studies were excluded as the researcher wanted the experiences which could be obtained in qualitative studies as they provided adequate information. The inclusion and exclusion criteria were included to match the studies conducted with undergraduate nursing students and professional nurses and record the factors that facilitate professional socialisation of learners in the clinical learning areas.

### Search outcome

The comprehensive search from MEDLINE, CINAHL, Ebscohost, Google Scholar and Science Direct produced a total of 3035 articles. The articles were categorised to assess if they met the selection criteria. The categories used were: population, research methodology and the period when the articles were published. Most were published before 2008, others were addressing medical students and some were quantitative studies. Table 1 illustrates the search outcome.

**TABLE 1:** The search outcome.

Data bases searched	MEDLINE, CINAHL, Google Scholar, Science Direct, Ebscohost
Number of articles retrieved	3035
Articles removed because of duplication	1505
Articles addressing other health professionals	1103
Articles removed because they were written in other languages and not English	55
Articles removed as they were quantitative studies	203
Articles published outside 2008–2018	561
Studies removed as they were abstracts only	65

The search produced 3035 results. After the review, 1505 articles were removed as they were duplicating. The remaining 1539 were reviewed, 1103 were found to be addressing other health professionals and not nursing learners. The remaining 434 articles were reviewed, 55 were written in other languages and the English version was not found, 65 of them were only abstracts and not the real studies, 203 were quantitative studies, the remaining 561 articles were published outside the period 2008–2018. After the selection process, 13 articles met the eligibility criteria and were included in this study. The full study selection process is illustrated in Doi: 10..

### Quality appraisal

The Critical Appraisal Skills Program (CASP) research checklist and the CASP (Singh 2013) were used to assess the quality of data. The appraisal is used to assist the researcher in assessing the published literature for trustworthiness (Singh 2013).

The papers were assessed for their clear indication of the aim of research, methodology and research design applied, ethical issues, sufficient data analysis, findings and the value of the research. The researcher first reviewed the articles and then sent them to two other researchers for their evaluation. Each of the researcher reviewed the articles separately and then came together to discuss the findings. Table 2 indicates the criteria used for assessing the quality of the studies.

**FIGURE 1:**
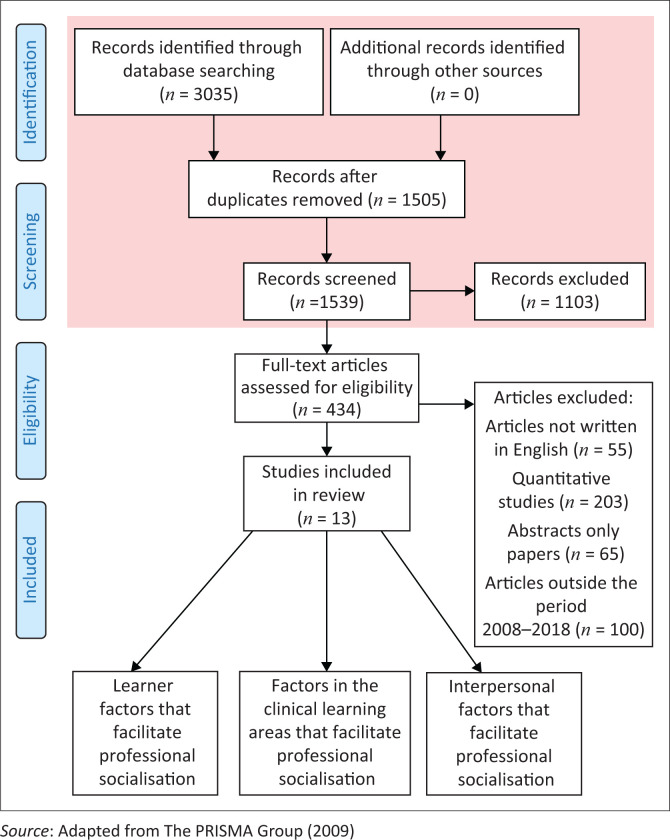
Flow chart depicting the literature search.

**TABLE 2:** Critical appraisal.

Critical appraisal skills program criteria	Criteria used
A clear statement of the aims of the research	The reviewed articles were assessed for the aim and objective of the research, significance and if the research was relevant. All articles were compliant.
Was the qualitative methodology used?	The reviewed literature used qualitative methodology and systematic review to collect data. All the methodologies used in all the studies were appropriate.
Was the research design appropriate to address the aims of the research?	All the designs used in the reviewed articles were appropriate and suitable for the research. The research designs were justified.
Was the recruitment strategy appropriate to the aims of the research?	The reviewed literature thoroughly explained and justified the selection and recruitment of participants.
Was the data collected in a way that addresses the research issue?	The literature explicitly indicated and justified the research setting, the data collection method, the role of the researcher during data collection and the duration thereof.
Was the relationship between the researcher and participants adequately considered?	The literature reviewed explained and justified the researcher as the instrument for data collection and the interaction between the researcher and the participants.
Have ethical issues been taken into consideration?	The reviewed literature thoroughly explained the ethical considerations. Participants’ rights to participate were explained and not violated. The permissions to conduct the research were obtained from different stakeholders concerned. Participants’ rights to privacy were protected.
Was the data analysis sufficiently rigorous?	Data analysis processes were explained, themes that emerged were discussed and sufficient data to support the findings were provided.
Was there a clear statement of findings?	Findings of the reviewed literature were explicit. Discussions were vigorous, the credibility of findings was discussed and the findings were discussed concerning the research questions. Literature was searched to support the findings.
Was the research valuable?	The reviewed literature addressed the objectives of the study, recommendations related to the study were indicated. Limitations of the studies were discussed.

*Source:* Singh, J., 2013, ‘Critical appraisal skills programme’, *Journal of Pharmacology and Pharmacotherapeutics* 4(1), 76.

### Data synthesis

This is the last stage in the review. It is the process of coding of findings reported by the primary studies (Siddaway 2014). The initial review identified 3035 research articles which were further reviewed according to the selection criteria. After screening, 1539 remained. After more synthesis, 13 articles which met the inclusion criteria were included in the analysis of the study. The synthesis had three stages whereby the researchers first analysed the findings from the reviewed literature. The findings were later reviewed by other two researchers who had extensive knowledge on systematic literature review. The findings were later compared and consolidated. The findings that were related to each other were clustered together and organised into themes and subthemes. Table 3 highlight the themes, sub-themes and the articles used to derive the themes and sub-themes.

**TABLE 3:** Themes, sub-themes, and references.

Themes	Sub themes	Sources
Learner factors that facilitate professional socialisation	Professional development and identity	Brown, Stevens & Kermode (2012) Melrose, Miller, Gordon & Janzen (2012) Zarshenas, Sharif, Molazem, Khayyer, Zare & Ebadi (2014) De Swardt, Van Rensburg & Oosthuizen (2017) Keeling & Templeman (2013)
	Motivation and attitude	De Swardt, Van Rensburg & Oosthuizen (2014) Kanyamura, Longwe, Haruzivishe, Kasu, Gwariro, Dzimiri et al. (2016) Zarshenas, Sharif, Molazem, Khayyer, Zare & Ebadi (2014) Salisu, Dehghani Nayeri, Yakubu & Ebrahimpour (2019)
Factors in the clinical learning area that facilitate professional socialisation	Clinical support and learning	De Swardt, Van Rensburg & Oosthuizen (2017) Habibzadeh, Ahmadi & Vanaki (2013) Hunter & Cook (2018)
	Values and belief system	Brown, Stevens & Kermode (2012) De Swardt, Van Rensburg & Oosthuizen (2017) Hunter & Cook (2018)
	Role modelling	Brown, Stevens & Kermode (2012) De Swardt, Van Rensburg & Oosthuizen (2017) Felstead & Springet (2016) Lúanaigh (2015) Zarshenas, Sharif, Molazem, Khayyer, Zare & Ebadi, A. (2014) Hunter & Cook (2018)
Interpersonal factors facilitating professional socialisation	Teamwork	Condon & Sharts-Hopko (2010) Lúanaigh (2015) Hunter & Cook (2018)
	Communication	Condon & Sharts-Hopko (2010) Habibzadeh, Ahmadi & Vanaki, Z. (2013) Hunter, K. & Cook (2018)

The themes that emerged from the review were as follows: (1) Learner factors that facilitate professional socialisation, (2) Factors in the clinical learning areas that facilitate professional socialisation, and (3) Interpersonal factors that facilitate professional socialisation.

The summary of the reviewed literature is indicated in Table 4, which presents the name of the author/s and the year of publication, the country, the samples, the sample size, the aim of the study and the findings of the study.

**TABLE 4:** Articles selected for review for the professional socialisation of learner nurses.

	Reference	Country	Sample	Sample size and data collection	Research aim	Findings
1.	Brown et al. (2012)	Australia	Newly graduated Registered nurses and clinical teacher	Semi-structured interview Seven clinical teachers and seven newly graduated learners	To develop an understanding of the role of the clinical teacher in the process of professional socialisation of student nurses as expressed/perceived by the clinical teacher and newly graduated registered nurses	Professional role concept or identity is affected by many factors in the clinical learning environment Clinical learning environment provide students with exposure to the nursing role allowing them to internalise values and norms Role modelling provides students with the opportunity to emulate the behaviour of nurses
2.	Condon and Sharts-Hopko (2010)	Japan	Nursing students	8 interviews	To explore the socialisation process experienced by Japan nursing students	Openness to accommodate others is significant to professionalisation of nursing students Effective communication in the clinical learning is in professional socialisation Team work in the clinical learning areas create an environment conducive for professional socialisation Feedback should be part of learning in the clinical learning areas to give students time to reflect on their performance
3.	De Swardt et al. (2014)	South Africa	Professional nurses and student nurses	14 professional nurses and 48 students Focus group interviews	To explore the perception of professional nurses regarding their role in the professional socialisation of student nurses and the experiences of the students of professional socialisation as members of the nursing profession	Students felt unsupported and not properly mentored Professional nurses viewing students as arrogant Professional nurses involved in misconduct in the presence of students Some students came to nursing because of other reasons not the core values of the profession
4.	De Swardt et al. (2017)	South Africa	Professional nurses and student nurses	Focus group	To develop and validate guidelines to support professional nurses and educators in the professional socialisation of student nurses	The significance of a positive clinical learning environment Educators to be role models to students The importance of shaping student’s behaviour at an early stage Fostering a professional identity
5.	Felstead and Springet (2016)	United Kingdom	Nursing students	12 face-to-face in-depth interviews	To explore the students’ lived experiences of role modelling through an interpretive phenomenological analysis approach aiming to understand the impact of their development as professional practitioners	Professional nurses have a strong influence on nursing students’ perception of role models and professional development Role modelling of good conduct by professional nurses is significant in student learning
6.	Habibzadeh et al. (2013)	Iran	Registered nurses	18 semi-structured interviews	To explore facilitators and obstacles to nursing professionalisation from Iranian nurses’ perspective	Communication in the clinical learning areas to be conducive to increase the gaining of better professional identity if students Students to be self-motivated and have a positive attitude in order to be properly socialised in the profession Organisational structures like human resource directly impact professional socialisation of students Students’ support in the clinical learning areas is significant towards professionalisation
7.	Kanyamura et al. (2016)	Zimbabwe	Learner nurses	Systematic review Nine articles	To explore and describe the concept of professional socialisation in nursing.	Attributes to professional socialisation Antecedents and consequences of professional socialisation Consequences of professional socialisation
8.	Keeling and Templeman(2013)	United Kingdom	Final year nursing students	10 focus group and semi-structured interviews	To explore final year nursing students’ perceptions using a reflective approach	Students frustrated by the attitudes of the society regarding their caring ability which affects their professional identity Behaviour of professional nurses significant to the learner in development of professional identity and socialisation
9.	Melrose et al. (2012)	Canada	Student nurses	27 face-to-face interviews and focus group	To describe student nurses’ experience with professional socialisation as they transitioned into a more complex role	Professional identity is important in professional socialisation Interactions including acceptance and acknowledgement by others in the clinical learning areas are key socialising agents
10.	Lúanaigh (2015)	Australia	Student nurses and registered nurses	Five students individual interview and focus group	Explore the influence of registered nurses on the nursing students’ learning in the clinical environment	Students learn effectively through interaction with others Students need to belong in order to interact and be professionally socialised Team work in the clinical learning environment supports professional socialisation Role modelling influences the professional identity of the students by providing positive examples of good nursing.
11.	Zarshenas et al. (2014)	Iran	Nursing students and registered nurses	43 students 8 registered nurses and focus group interviews	To increase the understanding of professional socialisation in nursing and explore the related factors from the perspective of registered nurses and nursing students	Sense of belonging can influence the process of professional socialisation Development of positive professional identity assist the student to understand the meaning of nursing and helps in professional socialisation. Students needs to have intrinsic motivation which helps to form a professional identity Role models in the clinical learning areas assist students in professional identity and socialisation
12.	Salisu et al. (2018)	Iran	Learner nurses and professional nurses	Systematic review Eight articles	Identify challenges of professional socialisation Identify facilitators of professional socialisation	Challenges of professional socialisation Professional factors Personal factors Educational factors Facilitators of professional socialisation Professional factors Personal factors Educational factors
13.	Hunter and Cook (2018)	New Zealand	Registered nurses	5 semi-structured face-to-face interviews	To explore new graduate nurses’ experience of professional socialisation by registered nurses in hospital based practice setting and to identify strategies that support professional identity development	Recognising positive role models and accessing clinical support of students Prioritising holistic ethical care with the use of clinical judgement, effective communication and patient advocacy Team support for confidence and a sense of belonging in the clinical learning environment

### Learner factors that facilitate professional socialisation

#### Professional development and identity

A positive professional development and identity lead to effective professional socialisation (De Swardt et al. 2017; Melrose et al. 2014). The perception of learners was that during clinical placement and training, they have changed from what they were before the allocation to their current status (Hao et al. 2014). The students expressed that the experiences helped them to develop professionally and to become aware of many professional issues in the profession (Habibzadeh et al. 2013; Keeling & Templeman 2013). The authors indicated that the students described that their journey led to them developing professionalism in the profession and they further described how the development of a nursing identity is an important factor in students’ success. Students and clinical teacher both indicated how the role of the clinical teacher is significant in supporting students to develop strong and positive nursing identities (Brown et al. 2012).

#### Motivation and attitude

The reviews indicated motivation as a contributor to professional socialisation. It is significant that a person must be motivated to be adequately socialised in the profession (Saeedi & Parvizy 2019; Zarshenas et al. 2014). It was further stated that when nurses are motivated they believe in themselves and feel free to be able to participate in the activities taking place in the units. The negative and positive attitudes that the student experienced during clinical placement might influence the outcome of professional socialisation (De Swardt et al. 2017). Students were also exposed to incidents of hostility and stereotyping from the professional nurses (De Swardt, Van Rensburg & Oosthuizen 2014) which might affect their motivation. The authors further stated that students indicated that this affects their ability to learn and as such, they become defensive and aggressive to protect themselves from personal attacks. In order for students to be able to adapt and adjust in the profession, they need to be motivated. Motivation can also be intrinsic whereby the learner needs to be self-motivated to learn (Zarshenas et al. 2014). The professional nurses indicated that learners were at times passive, and not taking responsibility for their learning. They further indicated that those who are motivated and interested to learn could show that by enquiring about activities, asking questions and assistance during patient care (De Swardt et al. 2014; Habibzadeh et al. 2013).

### Factors in the clinical learning area that facilitate professional socialisation

#### Clinical support and learning

The reviews indicated that students needed support from experienced staff so that they can access new knowledge needed for the development of skills in the clinical learning area (De Swardt et al. 2014; Habibzadeh et al. 2013). It was further revealed that students working with professional nurses made them to be aware of different professional behaviours and attributes that they could then emulate (Keeling & Templeman 2013; Salisu et al. 2018). The support was expected from the professional nurses and nurse managers in the institutions as they are the people who are with the learner nurses during clinical placement (Kanyamura et al. 2016).

### Values and belief system

The reviewed literature alluded to the violation of ethical codes where patients were physically abused, students were humiliated and exploited, and patients’ dignity was ignored (De Swardt et al. 2014). The situations where patients were not allowed to make an informed decision, or not enabled to participate actively in their own care were identified (Brown et al. 2012; Kanyamura et al. 2016). It was further revealed that when professional nurses uphold the moral and ethical codes of the profession, students tend to internalise those behaviours through observing them in action (De Swardt et al. 2014; Zarshenas et al. 2014). This will help the student to develop self-concept and social identity within the profession which is consistent with and accepted by other members of the profession.

### Role modelling

Students view professional nurses as their role models (Brown et al. 2012). The effect of professional nurses’ role modelling further supports the Social Learning Theory which stated that young people emulate the behaviour of the adult people in their environment (David 2015). The reviewed literature revealed that students indicated that they have observed situations wherein professional nurses behave in such a manner that they would not want to behave (De Swardt et al. 2017; Felstead & Springet 2016). Professional nurses need to behave in a manner that will not bring the profession into disrepute. When professional nurses and clinical staff behave as exemplary role models in the clinical learning areas, students’ attitudes towards nursing were perceived to be more positively shaped (Brown et al. 2012; Hunter & Cook 2018; Lúanaigh 2015; Salisu et al. 2018).

### Interpersonal factors facilitating professional socialisation

#### Teamwork

Teamwork is not only significant in the professional socialisation of learners but for nursing practice also. The reviewed literature indicated the significance of teamwork among all professionals and learner nurses in the clinical learning environment as very important (Kaiser & Westers 2018). When learners become part of the team of other health professionals like doctors, physiotherapists, pharmacists, and so on, they feel relaxed and this makes them eager to learn (Condon & Sharts-Hopko 2010). The studies revealed that learners need to be considered as members of the health team and be involved in all the activities in the clinical learning areas including meetings, depending on the level of training (Habibzadeh et al. 2013; Salisu et al. 2018). The literature stated that learners should be part of the decision-making team regarding patient care and unit management in the clinical learning areas. Working as a team makes students to feel that they are part of the group and when they feel that they belong to the team, they can learn. It was stated that students experienced working with patients and families to be a challenge. It was further indicated that students found accommodating the behaviour of colleagues to facilitate teamwork to be a significant task (Habibzadeh et al. 2013; Hunter & Cook 2018).

#### Communication

Literature review revealed that communication between nursing education staff, clinical staff and learners and the entire health team promotes effective professional socialisation of nursing learners (De Swardt et al. 2014). In the studies, learners indicated that in the clinical learning areas there is support, but not adequate because some professional nurses at some instances fail to provide solutions to the questions posed by learners regarding patient’s conditions. Learners further stated that the Nursing Education Institution (NEI) staff do not come often to offer them support in the clinical learning areas (De Swardt et al. 2014). Students stated that at times they are not given enough time to study before tests as the NEI’s and clinical learning areas fail to communicate to arrange for students to be given time to prepare for the test.

Learners also indicated that they were taught how to properly communicate in the clinical areas. They indicated that proper communication should be considered as important in fulfilling professional role (Habibzadeh et al. 2013). Students indicated that communication increased the possibility of gaining a better professional identity and is an important tool for professional socialisation. Students further stated that communication should include the patient and other members of the health team (Hunter & Cook 2018; Lúanaigh 2015; Melrose et al. 2014).

## Discussion

The findings suggested that learner factors, factors in the clinical learning areas and interpersonal factors influence professional socialisation of learner nurses. The first to influence professional socialisation was a professional identity which was influenced by the learner’s motivation and self-esteem. The learner who had positive self-esteem found it easy to adapt in the clinical areas (Graham et al. 2016). Hao et al. 2014). Positive professional identity is the core of professional socialisation as the learner who is confident in practice would be motivated to learn and complete the training (Baldwin et al. 2017). Therefore, a learner who was motivated found it easier to adapt to the expectations of the profession, thus making professional socialisation more effective. A learner with positive professional identity can handle the stress in the clinical learning areas. The learner will be able to focus on the main objective because of the intrinsic motivation developed during professional identity. This is supported by the findings in the study by Sun et al. (2016) which revealed that professional identity is an internal incentive factor of individual career development and has the strongest impact on the nursing students’ level of role stress.

Professional identity includes professional, educational and social values, and is essentially perceived as what makes a person a professional and distinguishes one profession from another. The development of a positive identity and motivation of the learner nurse, nature of the clinical learning environment and the conduct of the professional nurses impact learner nurses’ professional socialisation. Guo et al. (2018) defined professional identity as the professional self or self-concept of nursing that represents how nurses or nursing students perceive the nursing profession.

Professional identity is part of the individual’s overall identity. Is it usually perceived as what makes the person be a professional? The formation of one’s identity in the profession is largely social and in relation to nature as it is influenced more by the hidden or informal curriculum that the formal teaching offered by academic institutions (McLean et al. 2015). The learner needs to develop a positive professional identity as this will influence the learning in the clinical learning areas. The first journey in professional socialisation is the development of professional identity and it includes transitioning into the chosen field.

Developing a professional identity is essential for students to form an impression of their chosen profession and acculturate into that profession. A positive professional identity can lead to personal, social and professional fulfilment; whereas a lack of professional identity may be a contributing factor in nursing students leaving the nursing programme, and graduate nurses leaving the profession (Guo et al. 2018) or poor professional socialisation leading to incompetency, malpractice and poor job satisfaction.

Learners begin to shape their professional identities even before they commence their training because before they choose the profession they have preconceived ideas about that profession. Studies conducted before indicated that there is a correlation between a strong cohesive professional identity and job satisfaction meaning that the learner who has developed a strong professional identity will be motivated and eager to learn making professional socialisation real (Browne et al. 2018). Learners come into the profession with different expectations and sometimes are disillusioned. The confusion can be worrisome if the professional nurses do not provide adequate support and guidance to the learners (Traynor & Buus 2016).

It is in clinical practice where learners acquire the skills and knowledge of the profession. Incidences of bullying, hostility and shaming were identified. At some other times, learners were left to fend for themselves without assistance from the professional nurses. The nature of the clinical learning environment and experiences can strongly influence the development of learner nurses’ professionalism in the nursing profession. In the clinical learning environment, professional identity might be negatively affected by hierarchical relationships and discrimination between the staff (Keshmiri et al. 2020).

The clinical learning staff should create an environment that is favourable for learning and professional socialisation. Some studies in North America have shown that the quality of the clinical learning environment that provided the context for training was a predictor of the quality of care provided by graduates for years after graduation (Nordquist et al. 2019). A more positive and supportive clinical learning environment promotes student learning and professional socialisation (Hashemiparast, Negarandeh & Theofanidis 2019; Porteous & Machin 2018). In the clinical learning area, an atmosphere should be created where students feel appreciated and supported in clinical practice by professional nurses and other members of the health team.

A clinical learning environment which involves a positive atmosphere, and a good interaction with clinical nurses, patients, and nurse teachers and patient care are appreciated by students (Teskereci & Boz 2019). Also, learners appreciate those clinical learning areas that enhance cooperation between the nurse teacher and staff nurses, ward atmosphere where they are treated like younger colleagues and nursing care which follows nursing philosophy that enhanced learning. This sentiment was also shared by D’Souza et al. (2015) who further stated that in such learning environments, the quality of student teaching, nursing and patient care improves. A positive clinical learning environment that offers positive interactions and support between the students and clinical staff is significant in promoting learning and professional socialisation contrary to the clinical environment where there are poor relations and absence of mutual respect (Anderson, Moxham & Broadbent 2016; Arkan, Ordin & Yılmaz 2018; Kuivila et al. 2020).

The clinical environment that promotes learning should create a situation where learners feel that they are part of the bigger health society and not excluded in matters of the unit. This is supported by Hegenbarth et al. (2015), in their study which indicated that the ideal learning environment that the units envision for their learners is characterised by openness, taking them underwing, and structuring to meet goals. To improve learning, a conducive learning environment is significant. The clinical environment should be friendly, learners should be treated fairly, clinical staff should not have unreasonable and improper expectations from them. Mistreatment of learners negatively impacts learning (Ashktorab et al. 2017).

Feedback to learners should be part of learning in the clinical environment. This will allow the learner to introspect and make corrections if necessary. Also, the students can be motivated to improve if they know about their level of performance. As stated by Sweet and Broadbent (2018), feedback is a well-known component of teaching and learning that can enhance and motivate a learner, or conversely intimidate and demotivate them depending on how it is given and received. The findings revealed inadequate communication between the nursing education institutions and the clinical learning areas.

A sense of belonging for learners in the clinical learning area is significant in promoting professional socialisation. Tirgari (2018) argued that if learners have a sense of belonging in the clinical areas, they appeared more comfortable in asking for advice and assistance when needed and could be involved in activities on the placement. Professional nurses in the clinical areas may promote a sense of belonging for learners by being friendly, enthusiastic and welcoming, respecting the learners as a person and team member, allowing the students to meaningfully contribute inpatient care, allowing them to engage in critical decision making in the units, and acknowledge and confirm the student’s proposed care (Perry, Henderson & Grealish 2018).

Belonging and acceptance are human needs, which are placed in the middle level of five tiers as described in Maslow and Lewis’s (1987) hierarchy of human needs. The need to be accepted is deep-seated, and the fear of rejection or exclusion can be demoralising to learners. Belonging as a nursing student impacts on the student’s ability, capacity, and motivation to learn and to make the most of their educational experiences as well as their ability to socialise on a professional basis and develop a professional identity (Maginnis 2018).

A study on nursing students, belongingness and workplace satisfaction (Borrott et al. 2016) revealed that there is a relationship between belongingness and workplace satisfaction. When learners form part of the health team and are included in the nursing activities like being given the responsibility to monitor patient care, tasks that encourage critical thinking and creativity, it builds their confidence and encourages them to learn more (Minton & Birks 2019). This makes the learner feel free to be part of the major health team and thus professional socialisation will be enhanced.

Role modelling was identified as a major factor that influences professional socialisation. Learners indicated witnessing inappropriate behaviours by professional nurses during patient care which they identified as being wrong. On the contrary, there were also positive role models whom the learners admired and wanted to emulate in future. To enhance professional socialisation, professional nurses should act as exemplary role models to learners. Role modelling is an ‘unintentional act of not only observing the behaviours of others but also interacting with other healthcare professionals’ (Kelly 2019). In Iran, learners had experiences where they had negative interpersonal relationships with clinical staff during placement. These led to learners feeling powerless, being excluded from the health team, not supported in teaching and being emotionally distressed. All these negatively impacted professional socialisation (Lee & Yang 2019).

Professional nurses need to demonstrate therapeutic communication, critical thinking, compassion, enthusiasm and positive attitudes all the time in the clinical areas. This is also supported by Gibbs and Kulig (2017) who indicated that learners described that the clinical instructors who in this case is the professional nurse, was an important factor in shaping their ability to think critically. De Swardt et al. (2017) in their study on supporting students in professional socialisation concluded that students consider nurses in the clinical field areas as the most influential role models in shaping their clinical practice, consequently their socialisation process. The studies further revealed that professional nurses in the clinical area who act as role models demonstrated good communication skills, positive attitudes, caring and excitement.

The presence of exemplary role models in nursing practice is very important as this kind of behaviour and attitude in the profession will empower the learners to have appropriate skills, knowledge, values and belief of the profession. Learners are inspired by professional nurses who have a positive attitude to challenging situations, who can multitask with diligence, who effectively communicate with the patient, members of the health team and manage to challenge clinical situations professionally. They consider such professional nurses to be role models and want to emulate such behaviour and conduct. The sentiment was also shared by Lyneham and Levett-Jones (2016) who indicated in their study that the clinical teachers, in this instance the professional nurses, implicitly acted as role models for the learners, enabling them to recognise the professional roles of nurses and shape their professional attitudes through a socialisation process.

In another study by Mendes, Da Cruz and Angelo (2015) students perceived that their instructors possessed both positive attitudes and a caring demeanour. The appearance of the professional nurse influences the image of the profession. Even how the professional nurses dress up in their uniform can influence the way the learner turns out to dress in the near future. The professional nurse’s role in influencing the professional socialisation of the learner does not only help the student to succeed in their training but also in their personal and professional journey to become a competent and skilful practitioner one who is caring and compassionate, who cares for people in a humanising way and one who has the resilience to challenge those who are not demonstrating humanised care (Jack, Hamshire & Chambers 2017; White et al. 2018).

In a situation where professional nurses act in a manner that does not uphold the moral and ethical principles of the profession, they are unintentionally sending the wrong message to the learners that it is ‘acceptable’ to act as such. This is supported by Paliadelis and Wood (2016) in their study wherein learners indicated that negative role models could have a positive influence on their practice and they demonstrated how reflections on negative events allowed them to re-imagine them more positively.

## Recommendations

To enhance the professional socialisation of learners in the clinical areas, the following recommendations were identified:

### The nursing education institutions

- Lay a foundation through the teaching of ethics and professional values before clinical placement.
- Ethics and professional conduct should be thoroughly deliberated with the learners for them to have information about the topic.
- Students should be encouraged to attend events where ethical conduct is discussed.
- Have constant communication with the clinical learning areas to discuss clinical issues that impact professional socialisation of learners.

### Professional and nurse managers in the clinical areas

- Professional nurses to act as exemplary role models as they are the ones who are always with the learners in the clinical learning areas.
- Managers in the clinical learning areas to offer support to professional nurses so they have the adequate resources to provide care to patients.
- The Nurses Pledge of service should be discussed often in the clinical learning areas to enhance internalisation and understanding of the code so that professional nurses and learners are reminded of their commitment.

## Limitations of the study

The primary limitation of this study was that the study was only about nursing learners whereas the concept of professional socialisation affects all professions. The other limitation was that the researcher reviewed the qualitative literature; quantitative literature could have also had valuable information about the phenomenon.

## Conclusion

To enhance professional socialisation, the learner must be motivated to be in the profession and must be taught about the accepted behaviours that the profession demands. The theory is always taught in the NEIs but applied in the clinical learning areas. The intrinsic motivation should drive the learner to want to be the nurse and develop the identity in the profession. A positive professional identity enhances socialisation. The clinical learning area must play its part in ensuring that the environment is conducive for learning. An environment which is supportive and where communication is effective will foster professional socialisation. Learners are like blank pages when they enter the clinical learning areas. The professional nurses are the authors of the professional socialisation, who write on these blank pages through role modelling and creating a conducive environment for learning in the clinical areas.
